# Compartmentalization of Free Fatty Acids in Blood-Feeding *Tabanus bovinus* Females

**DOI:** 10.3390/insects16070696

**Published:** 2025-07-06

**Authors:** Mikołaj Drozdowski, Mieczysława Irena Boguś

**Affiliations:** Museum and Institute of Zoology, Polish Academy of Sciences, ul. Twarda 51/55, 00-818 Warszawa, Poland; mikolajdrozdowski@gmail.com

**Keywords:** *Tabanus bovinus*, cuticle, free fatty acids, GC/MS

## Abstract

Lipids play vital roles in insect physiology, functioning as energy reserves, membrane constituents, and cuticular protectants. However, few studies have examined the anatomical distribution of lipids in blood-feeding Diptera and compared the compositions of the cuticular and internal compartments. This study analyzes the qualitative and quantitative profiles of free fatty acids (FFAs) in the female *Tabanus bovinus*, a hematophagous horsefly species, across different anatomical regions, including the head, wings, legs, thorax, and abdomen. The surface and internal lipid fractions were isolated using petroleum ether/dichloromethane extraction followed by sonication. GC-MS revealed the presence of 21 FFAs, including 16 saturated (C7:0, C8:0, C9:0, C10:0, C11:0, C12:0, C13:0, C14:0, C15:0, C16:0, C17:0, C18:0, C19:0, C20:0, C22:0, C24:0) and five unsaturated (C16:1, C18:2, C18:1, C20:5, C20:4). The head and wings showed the highest concentrations of cuticular FFAs. At the same time, internal lipid stores were most prominent in the thorax and abdomen (but four times lower than in the head cuticle), reflecting their role in energy storage and reproduction. All cuticular and internal extracts were dominated by C16:0, C18:0, and C18:1. Notably, several FFAs were undetected in specific compartments: C10:0 from inside the head, C11:0 and C13:0 from inside all examined body parts, C19:0 was absent from inside the head, wings and legs, while C20:5 and C20:4 were absent from both the cuticular and internal lipid pools of the wings. Interestingly, our analysis of the cuticle on the thorax and abdomen together revealed that both C13:0 and C19:0 were present only on the dorsal side, i.e., absent from the ventral side. These absences suggest a selective lipid metabolism tailored to the functional and ecological demands of *T. bovinus* females. Our findings suggest that the absence of specific compounds from individual body parts may serve as an indicator of physiological specialization. This work provides new insights into lipid compartmentalization in Tabanidae and offers a framework for future comparative and ecological lipidomics studies in insects.

## Simple Summary

Free fatty acids (FFAs) occupy unequal spatial distributions in the cuticle and internal lipid pool of female *Tabanus bovinus* Linnaeus (pale giant horsefly; Diptera: Tabanidae). Our findings indicate the highest concentrations of cuticular FFAs in the head and wings, and of internal lipid stores in the thorax and abdomen. In all extracts, C16:0, C18:0, and C18:1 were the dominant fatty acids. Several FFAs were absent from certain body compartments: C10:0 from inside the head, C19:0 from inside the head, wings, and legs, and C11:0 and C13:0 from the interiors of all examined body parts, while C20:5 and C20:4 were absent from both the cuticular and internal lipid pools in the wings. Cuticular C13:0 and C19:0 were detected only on the dorsal side of the thorax and abdomen. The uneven spatial distribution of FFAs suggests the existence of a selective lipid metabolism tailored to the functional and ecological demands of the insect.

## 1. Introduction

The rigid exoskeleton plays a crucial role in the life of insects, protecting their internal organs from mechanical injuries and water loss, and providing structural support to maintain the insect’s shape. Its flexible joints also enable efficient movement. The architecture of the exoskeleton is spatially diversified: it is characterized by varying properties, such as thickness and structure, depending on the body part [1,2,3]

A key component of the exoskeleton is the cuticle, i.e., the layer running around the outside surface; however, despite extensive research on its structure, little is known about the spatial differentiation of its physicochemical properties [4,5]. The cuticle serves as the primary level of protection against adverse environmental conditions, such as dehydration in terrestrial environments and excessive water influx in aquatic environments. Additionally, the cuticle safeguards against the harmful effects of UV radiation and acts as the initial protective barrier against numerous pathogens and xenobiotics present in the environment [5,6,7,8,9,10,11,12,13,14]. It could be argued that the cuticle, with its immense structural, morphological, chemical, and functional diversity, is one of the primary elements that have contributed to the evolutionary success of insects, allowing them to inhabit nearly all environments on Earth [15,16,17].

Despite its great species-specific morphological diversity, all cuticles are constructed according to a common plan. The structure consists of the epicuticle, the outermost thin layer covered with lipids; this prevents the passage of water and serves as protection against adverse external factors. Below the epicuticle lies the procuticle, composed of two layers: the exocuticle, which ensures mechanical protection through the presence of chitin and sclerotized proteins, and the elastic endocuticle, which is rich in chitin. The innermost layer is the basal layer. All layers of the cuticle are produced by epidermal cells [18,19,20].

The cuticle is additionally reinforced with various chemical substances that enhance its resistance to damage, enabling insects to survive in extreme conditions [21]. The structure and detailed composition of the cuticle can vary depending on the insect species, habitat, lifestyle, as well as developmental stage, sex, and physiological state [19,20,21,22]. Epicuticular lipids play a particularly important role in the survival of individual organisms in the face of threats, as well as in ensuring the continuity of the species [23,24].

Lipids play indispensable roles in the physiology, ecology, and evolution of insects. These compounds serve not only as metabolic fuels and building blocks of cellular membranes but also as mediators of environmental interactions, including chemical signaling, water balance, and defense [23,24,25,26]. Among the most functionally diverse lipid pools in insects are those of cuticular and internal lipids, whose compositions reflect both ecological adaptations and life history traits [27,28].

Cuticular lipids, predominantly composed of hydrocarbons and free fatty acids (FFAs), form a hydrophobic barrier that minimizes transcuticular water loss and provides protection against microbial pathogens and environmental stressors [23,28,29]. These lipids also play a role in inter- and intraspecific communication, particularly in species relying on chemical cues for mate recognition or territoriality [30]. The cuticular lipid composition is often highly species-specific and ecologically regulated, with desert-dwelling insects, for example, accumulating long-chain saturated hydrocarbons that improve cuticular impermeability [31]. The composition and thickness of the epicuticular lipid layer vary across species and developmental stages, reflecting functional specialization and environmental pressures [30,32].

In contrast, internal lipids are primarily associated with energy storage and metabolic regulation. The fat body of insects, functionally analogous to vertebrate liver and adipose tissue, stores lipids in the form of triacylglycerols and mobilizes them during energetically expensive processes such as flight, molting, and reproduction [27,33]. In such cases, triacylglycerols stored in the fat body are hydrolyzed into FFAs and transported via hemolymph to the flight muscles, where they are oxidized in mitochondria to meet their high metabolic demands [23,25,34]. This process is tightly regulated by endocrine factors such as adipokinetic hormone (AKH), underscoring the integration of the lipid system with hormonal control networks [35,36]. Additionally, internal FFAs serve as precursors for eicosanoids, hormone-like signaling molecules involved in immunity, reproduction, and development [23,37]. Lipids contribute significantly to the structural and functional integrity of insect tissues. In particular, free fatty acids and hydrocarbons incorporated into cell membranes modulate membrane fluidity, permeability, and protein function [38,39]. The fatty acid composition of membranes can shift in response to environmental temperature, a phenomenon known as homeoviscous adaptation, which allows ectothermic insects to maintain cellular function across thermal gradients [40,41].

Lipid metabolism plays a crucial role in reproduction by supporting egg production and gonadotrophic cycles in bloodsucking insects [42,43,44]. Among the Diptera, the members of the *Tabanidae* (horsefly) family are of special interest due to their blood-feeding behavior, strong flight capacity, and pronounced sexual dimorphism. Female horseflies require a blood meal to initiate vitellogenesis, during which lipids are mobilized to the developing oocytes [45]. Despite the known importance of lipids, many gaps remain in our understanding of their anatomical distribution and functional partitioning in non-model insects. While numerous studies have characterized lipid content in whole-body extracts or fat bodies, few have differentiated between external (cuticular) and internal (metabolic) lipids. Even fewer studies have analyzed how these lipids vary among distinct anatomical regions, such as the head, wings, legs, thorax, and abdomen, each with unique structural and functional properties.

*T. bovinus* is a widespread Palearctic horsefly species whose females exhibit strong hematophagy and high reproductive investment. As an active diurnal flier with specific seasonal activity, it is likely to possess lipid adaptations linked to energy demand, thermoregulation, and environmental stress resistance. These demands likely require not only efficient energy storage mechanisms but also a finely tuned balance between barrier protection (cuticle) and metabolic readiness (internal lipids). Investigating the spatial distribution of lipids in this species provides an opportunity to better understand how lipid metabolism and allocation contribute to the ecological and physiological success of hematophagous Diptera.

This study sets out to characterize the anatomical distribution of free fatty acids in female *T. bovinus*, with a focus on cuticular and internal lipids. By separately extracting surface and internal lipids, it was possible to compare the two sets of lipid profiles in the different regions of the main body: the head, wings, and legs, and the thorax and abdomen combined. Particular attention was paid to the absence of specific FFAs from certain body regions, which may indicate spatial metabolic selectivity. This work contributes to a broader understanding of lipid biochemistry in flies and provides a foundation for future studies on energy allocation, ecological physiology, and adaptations in lipid metabolism of blood-feeding insects.

## 2. Materials and Methods

### 2.1. Insects

*T. bovinus* females were caught while attacking humans in a rural area (Rudenka, 49.486555, 22.412092). The area was part of the Natura 2000 European Ecological Network, located in a sparsely populated region 30 km from the Polish-Ukrainian border, where no farm animals have been kept for at least 10 years. After being caught, the females were immediately frozen at −20 °C. Before lipid extraction, the frozen insects were measured and weighed.

### 2.2. Extraction of Free Fatty Acids (FFAs)

The cuticle and internal lipid components were extracted from various body parts (head, wings, legs, thorax, and abdomen) of three adult female *T. bovinus*. In *T. bovinus,* it is not possible to separate the thorax from the abdomen without damaging the body. The body parts were separated, weighed, and individually extracted in 20 mL of petroleum ether (Merck, Darmstadt, Germany) for five minutes (extract I) and then again in 20 mL of dichloromethane (Merck, Darmstadt, Germany) for another five minutes (extract II). These two extracts (I and II) containing the cuticular lipids were combined and evaporated in a stream of nitrogen. The use of petroleum ether minimizes the possible extraction of internal lipids [32]. The insect body parts were sonicated each in 20 mL of dichloromethane for one minute to liberate the internal lipids (extract III). Each extract was placed in a separate glass flask, evaporated under nitrogen, and weighed.

Three thorax-abdomens were taken for analysis of the cuticles of the dorsal and ventral sides. Briefly, each part was washed with cotton swabs soaked in petroleum ether, followed by swabs soaked in dichloromethane. Each wash (petroleum ether dorsal, petroleum ether ventral, dichloromethane dorsal, dichloromethane ventral) used three cotton swabs. Each swab was left for 24 h in 10 mL of petroleum ether or dichloromethane to extract the lipids. After this time, the dorsal extracts (petroleum ether and dichloromethane) were combined, and the ventral extracts were combined; these were then evaporated under a stream of nitrogen, weighed, and used for chromatographic analysis. Another set of cotton swabs soaked in petroleum ether or dichloromethane was prepared as controls: these were not used for cuticle washing.

### 2.3. Derivatization Method

Each sample and 20 µL of internal standard (19-methylarachidic acid; 1 mg/mL; Merck, Darmstadt, Germany) were silylated with 200 µL of N,O-Bis(trimethylsilyl) trifluoroacetamide (BSTFA): chlorotrimethylsilane (TMCS) (99:1) (Merck, Darmstadt, Germany) mixture for one hour at 100 °C to obtain trimethylsilyl esters (TMS) of FFAs. The TMS values of the fatty acids were then analyzed by GC–MS. The internal standard (IS) for the GC-MS analysis was 19-methylarachidic acid; this compound separates well from all the sample constituents and was not previously present in the insect samples [46].

### 2.4. GC–MS Analyses

The GC–MS analyses were carried out on a GCMS-QP2010 system with a mass detector (Shimadzu, Kyoto, Japan) and a ZB-5 MS (Zebron, Phenomenex, Torrance, CA, USA) column (thickness 0.25 µm, length 60 m, diameter 0.25 µm). Helium was used as the carrier gas at a column head pressure of 112.1 kPa. The column oven temperature cycle was maintained at 50 °C for 3 min, then ramped up from 50 to 310 °C at a rate of 2 °C/min; the final temperature was then held for 10 min. The ion source temperature was 200 °C, and the interface temperature was 310 °C. Split mode was used with a split ratio of 10. All compounds were identified based on their TMS derivative fragmentation patterns and mass-to-charge ratios, as listed in the NIST 12 library.

The mass spectra of the fatty acid trimethylsilyl esters comprised M^+^ (molecular ion), [M-15]^+^, and fragment ions at *m*/*z* 117, 129, 132, and 145. The content of the compounds in the analyzed samples was calculated from the peak areas of the chromatogram. Each sample was analyzed in triplicate, and the results were expressed as mean and standard deviation. Response factors of one were assumed for all constituents. The GC-MS procedure is described in more detail elsewhere [47,48]

### 2.5. Statistics

The findings were analyzed using one-way analysis of variance (ANOVA) and a *t*-test. Tukey’s test was used for post hoc analysis. Each test was performed separately. All analyses were performed using Statistica 6 software (StatSoft Polska, Kraków, Poland). Differences were significant at *p* < 0.05.

## 3. Results

### 3.1. Appearance of T. bovinus Females

All *T. bovinus* females used in the study were large, heavy-looking flies with a stocky body and a characteristic convex head with large eyes separated by a narrow forehead. Their bodies were brown, with a distinctive pattern, measuring 2.53 ± 0.31 cm in length. The wings were transparent and wide, with small halteres located between the second and third segments of the thorax. The mouthparts were cutting-licking. The total weight of each insect ranged from 129 to 145 mg (mean 138.97 ± 6.96 mg). Among the body parts (Table 1), the heaviest was the thorax connected with the abdomen (115.61 ± 5.78 mg; constituting 83.19 ± 0.20% of total body mass), followed by the head (10.03 ± 0.88 mg; 7.20 ± 0.31% bm), a pair of wings (3.10 ± 0.08 mg; 2.68 ± 0.07% bm) and three pairs of legs (7.99 ± 1.06 mg; 6.88 ± 0.59% bm).

### 3.2. Extracts from T. bovinus Females

The highest mass of cuticular lipids was obtained from the head extract (4.46 ± 2.95 mg), and internal lipids from the thoraxes-abdomens (4.01 ± 0.29 mg). Similar extraction efficiency was noted with regard to insect body weight: the highest mass of cuticular lipids from the heads was 32.84 ± 18.45 µg extract/mg body weight, and the highest of internal lipids from thoraxes-abdomens was 28.99 ± 2.90 µg extract/mg body weight. However, with regard to the mass of the individual body parts, the highest yields were noted for the wings: 704.75 ± 141.31 µg extract/mg body part weight (cuticular lipids) and 337 ± 48.18 µg extract/mg body part weight (internal lipids). The lowest yields were obtained from the thoraxes-abdomens: 19.48 ± 13.65 µg extract/mg body part weight (cuticular) and 34.84 ± 4.24 µg extract/mg body part weight (internal). Interestingly, in contrast to the other body parts, the wings yielded significantly more cuticular lipids than internal lipids, both with regard to total body mass and the mass of the individual body part. No significant differences between cuticular and internal extracts were noted for the other body parts. No significant difference in cuticular lipid content was noted between the different cotton swab washes for the dorsal and ventral parts of the thorax-abdomen (Table 1). No lipids were extracted from control cotton swabs.

### 3.3. Free Fatty Acid (FFA) Content in Cuticular and Internal Lipids of T. bovinus Females

**Figure 1:**
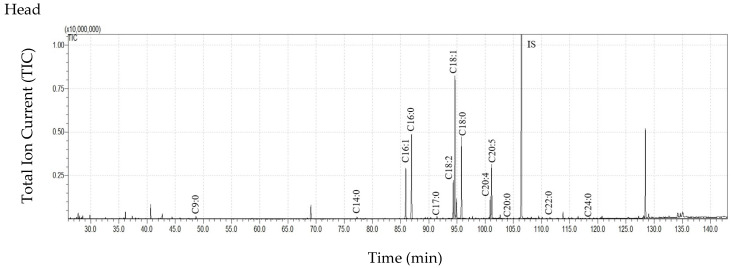

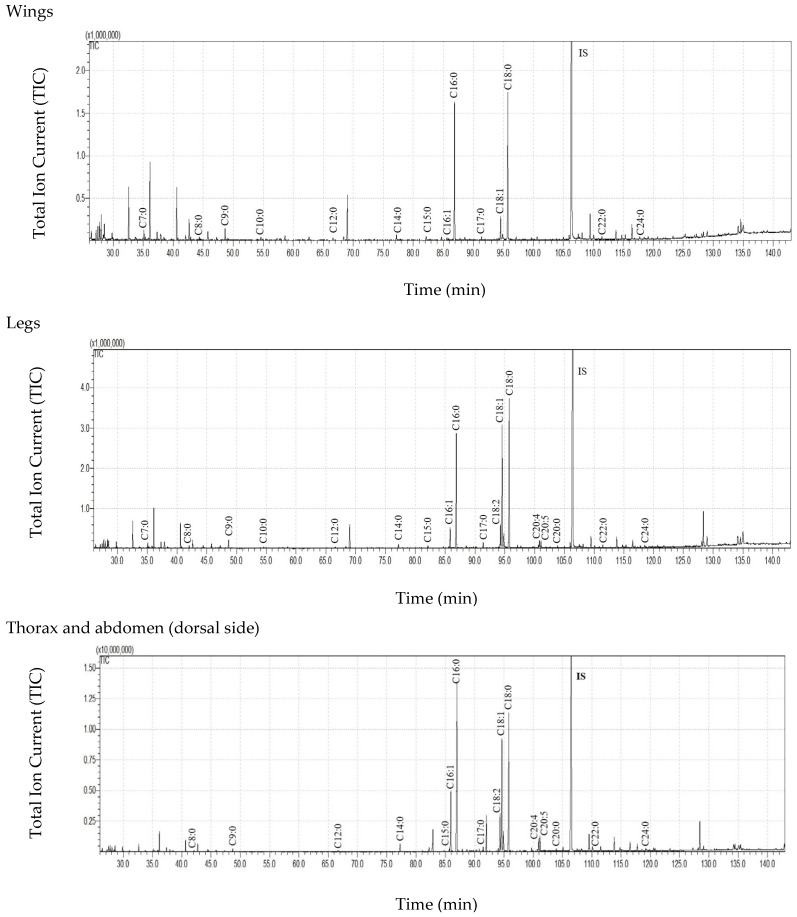

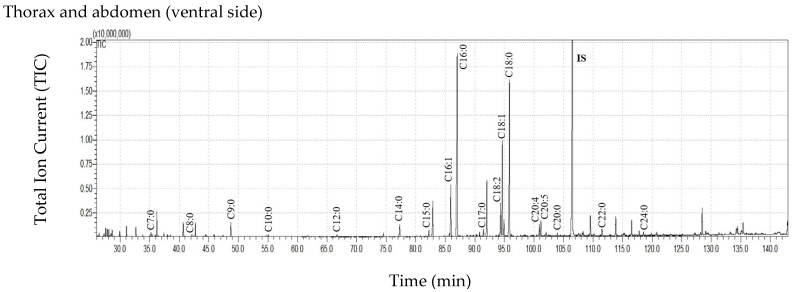
The total ion current (TIC) chromatogram of fatty acids (TMS esters) extracted from the cuticle of *Tabanus bovinus* females. Internal standard (IS, 19-methylarachidic acid); fatty acids and molecular ions: heptanoic acid (C7:0, *m*/*z* = 202), octanoic acid (C8:0, *m*/*z* = 216), nonanoic acid (C9:0, *m*/*z* = 230), decanoic acid (C10:0, *m*/*z* = 244), undecanoic acid (C11:0, *m*/*z* = 258), dodecanoic acid (C12:0, *m*/*z* = 272), tridecanoic acid (C13:0, *m*/*z* = 286), tetradecanoic acid (C14:0, *m*/*z* = 300), pentadecanoic acid (C15:0, *m*/*z* = 314), hexadecenoic acid (C16:1, *m*/*z* = 326), hexadecanoic acid (C16:0, *m*/*z* = 328), heptadecanoic acid (C17:0, *m*/*z* = 342), octadecadienoic acid (C18:2, *m*/*z* = 352), octadecenoic acid (C18:1, *m*/*z* = 354), octadecanoic acid (C18:0, *m*/*z* = 356), nonadecanoic acid (C19:0, *m*/*z* = 370), eicosapentaenoic acid (C20:5, *m*/*z* = 374), eicosatetraenoic acid (C20:4, *m*/*z* = 376), eicosanoic acid (C20:0, *m*/*z* = 384), docosanoic acid (C22:0, *m*/*z* = 412), tetracosanoic acid (C24:0, *m*/*z* = 440).

GC-MS analysis of the obtained extracts revealed the presence of 21 FFAs, including 16 saturated (C7:0, C8:0, C9:0, C10:0, C11:0, C12:0, C13:0, C14:0, C15:0, C16:0, C17:0, C18:0, C19:0, C20:0, C22:0, C24:0) and five unsaturated forms (C16:1, C18:2, C18:1, C20:5, C20:4). The total ion current (TIC) of the trimethylsilyl esters (TMS) of FFAs (after derivatization) extracted from the cuticles of various body parts are presented in Figure 1. The quantitative composition of the FFAs from all extracts is presented in Table 2. Among the individual FFAs, C16:0, C18:0, and C18:1 dominated in all samples. While the concentrations of the remaining FFAs differed between individual body parts, these differences were statistically insignificant due to the large scatter caused by individual variation.

The absence of two unsaturated FFAs (C20:4 and C20:5) from the cuticular and internal wing extracts is intriguing because both are present in all extracts from the other body parts. In addition, significant qualitative differences were noted between the cuticular and internal extracts: C11:0 and C13:0 were absent from all internal lipids extracted from all tested body parts. Also, C10:0 and C19:0 were missing from the internal extracts of the head, and C19:0 from those of the wing and leg (Table 2).

### 3.4. Cuticular Lipids of the Dorsal and the Ventral Sides of the Thorax and Abdomen

Due to the large size of the thorax-abdomen fragment, it was possible to examine more subtle differences in the spatial distribution of individual FFAs in its cuticle. In all extracts, C16:0, C18:1, and C18:0 were the dominant components. Also, while no significant differences in FFA concentrations were noted between the ventral and dorsal side cuticles, C13:0 and C19:0 were absent from the ventral side (Table 3).

## 4. Discussion

This study provides a comprehensive analysis of the composition of cuticular and internal lipids in different body parts of *T. bovinus* females. Our findings indicate that the external, i.e., cuticular, and internal compartments have distinct lipid profiles, and that these profiles vary between anatomical regions, reflecting their physiological and ecological adaptations.

The highest concentrations of cuticular lipids were observed in the head and wings, which may be linked to their functional exposure to the environment. The large head of *T. bovinus* (accounting for 7.2% of the total body mass) is equipped with a number of morphologically diverse and specialized organs, such as cutting-licking mouthparts, large compound eyes, antennae, and other sensory structures involved in host detection [49,50]. In Diptera, as in other insects, the fatty acids of the cuticle play a pivotal role in desiccation resistance, chemical communication, and defense against pathogens [28]. It is not surprising then that high levels of long-chain saturated and monounsaturated fatty acids (e.g., C16:0, C18:0, C18:1) were observed in the cuticle of the head; these could have waterproofing functions, which are particularly crucial during blood-feeding behavior when the insect is exposed to host defense mechanisms and environmental stress [29]. Similarly, C16:0, C18:0, and C18:1 were also found to dominate in a closely related species, *T. bivittatus*, as well as various other unrelated insect species [51,52,53,54,55,56]; this is not surprising as these FFAs are the most widely distributed and abundant fatty acids in nature [57,58,59].

The detection of the bioactive polyunsaturated fatty acids (PUFAs) C20:4 and C20:5 in the internal lipids of the head may indicate high local demand, perhaps to support neuronal signaling and synaptic plasticity [60]. Their presence inside the legs, thorax, and abdomen is also physiologically justified because they are key precursors of eicosanoids, a class of signaling molecules that play vital roles in insect immunity and reproduction [23,38]. However, it is more difficult to understand why they were present in the cuticle of all studied body parts, except the wings. Although both PUFAs have been detected in the cuticle of many other insect species [61,62,63,64,65], their physiological significance remains unclear. They may play a role in antimicrobial protection against entomopathogens; indeed, several saturated and unsaturated FFAs, including C20:5 and C20:4, have demonstrated remarkable antibacterial properties towards both Gram-positive and Gram-negative bacteria [66,67,68,69], although further research is required. In turn, the absence of C20:4 and C20:5 from the internal and cuticular extracts in the wings seems logical. By limiting the presence of such oxidatively unstable PUFAs in the wings, *T. bovinus* may enhance its resistance to environmental stressors and reduce oxidative stress in highly active tissues, such as flight muscles. This would support the theory that cuticular lipid composition is optimized to ensure structural stability and defensive efficacy [70]. Due to the lack of studies on the presence of PUFAs in the wings of other insect species, it is not known whether the lack of C20:5 and C20:4 in the wings despite their presence in other parts of the insect body is a common phenomenon in the insect world or whether it is limited to *T. bovinus* females.

Despite their light weight, the wings achieved a surprisingly high yield of extractable lipids per milligram of tissue. This may reflect their large surface-to-volume ratio and the presence of lipid-rich tracheal structures, which are involved in flight [30].

Interestingly, the internal lipid reserves were quite evenly distributed between different body parts, suggesting that their amount is limited, and no individual body part functions as the main lipid reservoir. Surprisingly, the mass of the extract obtained from the sonicated thorax-abdomen section was relatively low compared to the mass of the body part (Table 1). A much higher yield was expected due to the presence of the fat body, which acts as a primary lipid storage site.

Due to the considerable variability present between individual insects, the obtained data were subject to large scatter. Furthermore, the insects were caught when attacking humans in field conditions, and the life history of the insects and their physiological status are unknown. It is also unknown whether the captured individuals were fertilized and in which phase of the gonadotrophic cycle they were. It is believed that *T. bovinus* females typically search for prey, mainly large mammals such as cattle and horses, only after the mating flight and fertilization by males, although attacks by unfertilized females have been recorded. It is also known that *T. bovinus* females can undergo two or three gonadotrophic cycles, with each gonadotrophic cycle lasting on average four to seven days; however, its length is strictly dependent on environmental conditions [49,71,72,73]. In blood-feeding insects, both fertilization and the gonadotrophic cycle phase are closely linked to changes in the hormonally regulated lipid metabolism necessary for egg production [74,75,76,77]. Hence, to more precisely determine the composition of cuticular and internal lipids, further studies using *T. bovinus* grown under controlled conditions are necessary.

Nevertheless, despite the considerable individual variability observed among the females used in the present study, significant differences were found between the cuticular and interior lipid profiles of the studied body parts. Most obviously, the three saturated FFAs C11:0, C13:0, and C19:0, which were present in the cuticles of all body parts, were absent from the internal lipid pool. Such a stark contrast between the cuticular and internal lipid profiles for all body parts illustrates the compartmentalization of lipids in *T. bovinus*. Cuticular FFAs are likely synthesized or transported locally to reinforce barrier function, whereas internal lipids are more dynamic and mobilized during physiological processes. A similar differentiation in lipid roles has been observed in other Diptera, including *Musca domestica* and *Drosophila melanogaster*, where internal lipids are closely tied to reproductive and metabolic states [27,78]. It is unclear why C10:0 is absent from the interior lipid pool of the head while it is present in all other cuticular and internal extracts.

The lipid composition of an insect is shaped by its environmental conditions, dietary intake, and evolutionary lineage. Aquatic insects are characterized by high proportions of long-chain PUFAs, such as C20:5, C20:4, and C18:3, and terrestrial species by high proportions of C18:2 [79]. In *D. melanogaster*, a clear shift from shorter to longer cuticular hydrocarbon chain length was observed with increasing temperature [80]. Insects from arid environments commonly demonstrate long-chain saturated FFAs such as C22:0 and C24:0, which contribute to water retention and cuticular impermeability [51,81]. While *T. bovinus* inhabits wetter environments, such as grasslands and forest edges, and hence is under less evolutionary pressure to synthesize and/or retain such lipids in the cuticle, our findings nevertheless confirm them to be present in all tested body parts, accompanied by abundant shorter-chain saturated, monounsaturated, and doubly unsaturated FFAs. The absence of C13:0 and C19:0 on the ventral side of the thorax-abdomen part, and their presence on the dorsal side, indicates clear compartmentalization of lipids between body regions; however, further research is needed to understand the physiological implications of this spatial separation.

From an ecological perspective, the lipid composition of *T. bovinus* may be further influenced by environmental factors, including temperature, humidity, and host availability. The presence of thermally stable saturated FFAs in the cuticle could reflect adaptation to the high field temperatures typically experienced by *Tabanus* spp, during their summer activity peak. Indeed, ectothermic insects have been found to adjust their lipid profiles seasonally or in response to thermal stress [29].

In conclusion, the fact that certain fatty acids were absent from particular extracts, particularly those from some of the cuticular compartments, reflects the specialized lipid metabolism of *T. bovinus*. These patterns appear to support ecological adaptations, energy demands, and functional compartmentalization. Future research may further investigate how environmental changes, developmental stages, or dietary shifts affect these lipid profiles among *Tabanidae* species.

## Figures and Tables

**Table 1:** Quantitative summary of the experiment: weights of *Tabanus bovinus* body parts used for lipid extraction and lipid yield.

Body Parts	Mass of Body Part (mg ± SD)	Extract Mass (mg ± SD)		Extract Mass per Total Insect Mass * (µg Extract/mg Body Weight ± SD)		Extract Mass per Mass of the Extracted Insect Body Part (µg Extract/mg Body Part ± SD)	
		Cuticicular Extracts	Internal Extracts	Cuticicular Extracts	Internal Extracts	Cuticular Extracts	Internal Extracts
Head	10.03 ± 0.88 ^a^	4.46 ± 2.95	1.99 ± 0.95	32.84 ± 18.45	14.67 ± 6.54 ^c^	461.70 ± 328.99	207.75 ± 124.91
Wings (one pair)	3.10 ± 0.08 ^ab^	2.19 ± 0.48 ^A^	1.04 ± 0.13 ^A^	15.73 ± 2.64 ^B^	7.55 ± 1.06 ^dB^	704.75 ± 141.31 ^efghC^	337.53 ± 48.18 ^lC^
Legs (three pairs)	7.99 ± 1.06 ^b^	1.99 ± 0.68	1.51 ± 1.84	14.55 ± 4.75	10.75 ± 10.52	263.25 ± 133.20 ^eijk^	184.64 ± 212.35
Thorax and abdomen	115.61 ± 5.78 ^a^	2.31 ± 1.65	4.01 ± 0.29	16.22 ± 11.40	28.99 ± 2.90 ^cd^	19.48 ± 13.65 ^fi^	34.84 ± 4.24 ^l^
Thorax and abdomen (dorsal side) **	NA	1.20 ± 0.82	NA	8.43 ± 4.54	NA	10.12 ± 6.69 ^gi^	NA
Thorax and abdomen (ventral side) **	NA	1.10 ± 0.99	NA	7.78 ± 5.63	NA	9.35 ± 8.25 ^hk^	NA

* The weight of *T. bovinus* females (N = 3) used in the experiment ranged from 129 to 145 mg (mean 138.97 ± 6.96 mg). ** Cuticular lipids from the dorsal and ventral parts of the thorax and abdomen were collected using cotton swabs as described in Materials and Methods. In *T. bovinus,* it is not possible to separate the thorax from the abdomen without damaging the body, nor is it possible to separate the ventral part from the dorsal part. Statistically significant differences (*t*-test, *p* < 0.05) are marked with the same letters: small letters (a–l) indicate differences between body parts, and capital letters (A–C) indicate differences between cuticular and internal extracts. SD—standard deviation, NA—not applicable.

**Table 2:** Qualitative and quantitative composition of fatty acids in the surface and internal lipids of *Tabanus bovinus* females.

FFA	Concentration of FFA (ng/mg Body Part ± SD)							
	Cuticular Lipids				Internal Lipids			
	Head	Wings	Legs	Thorax and Abdomen	Head	Wings	Legs	Thorax and Abdomen
C7:0	192.79 ± 261.79	122.50 ± 59.80	57.28 ± 33.80	6.40 ± 3.56	4.68 ± 6.37	29.29 ± 12.87	19.75 ± 17.37	1.55 ± 1.35
C8:0	263.32 ± 318.50	225.83 ± 88.10	110.30 ± 67.59	11.21 ± 6.25	44.80 ± 46.88	57.34 ± 22.30	38.83 ± 34.67	8.78 ± 4.75
C9:0	491.46 ± 426.78	568.06 ± 143.54	255.11 ± 84.82	57.23 ± 38.45	92.60 ± 67.98	161.77 ± 27.75	116.90 ± 112.64	14.08 ± 2.66
C10:0	189.13 ± 255.43	93.50 ± 8.62	67.06 ± 37.51	9.08 ± 6.65	ND	13.50 ± 13.01	16.86 ± 23.72	1.16 ± 1.01
C11:0	108.42 ± 165.98	24.42 ± 27.73	11.50 ± 19.92	0.78 ± 0.57	ND	ND	ND	ND
C12:0	165.40 ± 163.32	128.76 ± 54.61	76.24 ± 41.23	12.55 ± 9.27	4.12 ± 7.14	20.16 ± 20.20	19.37 ± 27.99	3.92 ± 1.00
C13:0	96.32 ± 135.89	40.30 ± 18.83	19.85 ± 34.38	0.62 ± 1.08	ND	ND	ND	ND
C14:0	376.85 ± 202.09	318.45 ± 95.29	287.05 ± 205.40	69.86 ± 44.23	36.24 ± 25.98	76.77 ± 22.35	58.20 ± 45.81	32.08 ± 9.60
C15:0	192.64 ± 103.96	192.00 ± 112.85	222.23 ± 275.74	31.35 ± 16.68	3.87 ± 6.70	21.01 ± 20.49	22.06 ± 30.23	5.66 ± 3.04
C16:1	2821.03 ± 2708.41	75.90 ± 67.80	263.78 ± 228.93	225.44 ± 27.09	256.12 ± 149.43	32.26 ± 31.44	217.91 ± 266.88	1351.70 ± 227.11
C16:0	8561.72 ± 4167.84	5700.88 ± 2333.89	3852.48 ± 3535.31	1462.81 ± 774.09	844.95 ± 482.17	1399.51 ± 487.45	1499.76 ± 1544.96	1306.80 ± 195.63
C17:0	505.07 ± 259.02	119.79 ± 129.57	192.87 ± 183.57	28.46 ± 17.36	27.99 ± 21.54	18.74 ± 18.21	43.02 ± 42.28	52.71 ± 45.91
C18:2	1822.09 ± 2065.72	66.34 ± 78.10	310.73 ± 211.23	139.64 ± 32.38	205.30 ± 165.42	58.39 ± 31.54	341.89 ± 372.72	1241.33 ± 199.41
C18:1	8824.73 ± 9740.00	880.82 ± 614.15	1690.09 ± 1168.86	554.00 ± 73.15	1112.93 ± 866.09	815.14 ± 302.08	1935.62 ± 1998.02	3245.52 ± 349.59
C18:0	7630.55 ± 3329.36	5179.97 ± 2973.85	3353.72 ± 3325.72	1157.58 ± 745.98	727.07 ± 503.24	946.88 ± 610.97	1384.60 ± 1623.10	562.69 ± 99.04
C19:0	310.39 ± 482.22	9.03 ± 15.64	14.91 ± 25.83	43.65 ± 15.81	ND	ND	ND	8.63 ± 5.62
C20:5	2135.89 ± 2770.37	ND	142.50 ± 85.46	16.34 ± 11.57	256.91 ± 249.18	ND	272.47 ± 327.16	583.30 ± 135.54
C20:4	886.83 ± 1111.07	ND	119. 06 ± 79.37	46.96 ± 8.44	89.79 ± 83.03	ND	197.77 ± 234.67	474.75 ± 165.56
C20:0	72.40 ± 63.07	76.12 ± 66.72	54.00 ± 76.13	38.63 ± 33.93	7.47 ± 11.21	18.69 ± 19.05	20.81 ± 27.58	11.06 ± 2.01
C22:0	57.88 ± 62.75	75.57 ± 73.98	42.05 ± 56.65	6.29 ± 5.45	13.73 ± 21.30	58.37 ± 56.42	44.49 ± 59.46	5.53 ± 0.90
C24:0	48.89 ± 47.41	65.58 ± 58.98	39.06 ± 61.74	9.94 ± 11.33	15.34 ± 24.74	50.77 ± 46.60	31.22 ± 36.53	2.95 ± 2.57
Sum of FFAs	35753.80 ± 23741.37 ^abcA^	13964.79 ± 6581.49 ^adB^	11181.88 ± 9582.56 ^b^	4147.93 ± 1290.02 ^cd^	3743.91 ± 2528.51 ^A^	3778.59 ± 1651.56 ^B^	6281.54 ± 6812.38	8917.00 ± 1426.60

All results were calculated as ng/mg of insect body part used for extraction. Statistically significant differences (ANOVA, Test HSD Tukey, *p* < 0.05) are marked with the same letters: small letters (a–d) indicate differences between body parts, and capital letters (A, B) indicate differences between cuticular and internal FFAs concentrations. FFA—free fatty acid, SD—standard deviation, ND—not detected.

**Table 3:** Qualitative and quantitative composition of fatty acids in the dorsal and ventral cuticle of the thorax and abdomen of female *T. bovinus*.

FFA	Concentration of FFA (ng/mg of Whole Thorax and Abdomen ± SD)	
	Thorax and Abdomen Dorsal Side	Thorax and Abdomen Ventral Side
C7:0	3.22 ± 3.00	3.17 ± 0.56
C8:0	5.10 ± 3.54	6.11 ± 2.72
C9:0	27.93 ± 34.31	29.30 ± 4.18
C10:0	5.04 ± 5.91	4.03 ± 0.75
C11:0	0.48 ± 0.83	0.30 ± 0.36
C12:0	6.78 ± 6.93	5.77 ± 2.34
C13:0	0.62 ± 1.08	ND
C14:0	38.87 ± 35.60	30.99 ± 8.65
C15:0	17.23 ± 13.30	14.12 ± 3.39
C16:1	129.25 ± 30.04	96.19 ± 3.31
C16:0	807.11 ± 554.77	655.70 ± 219.98
C17:0	15.39 ± 13.23	13.07 ± 4.13
C18:2	92.11 ± 47.97	47.53 ± 15.62
C18:1	337.76 ± 216.25	216.25 ± 44.19
C18:0	633.63 ± 516.18	523.96 ± 230.15
C19:0	30.11 ± 18.57	ND
C20:5	9.17 ± 9.40	15.91 ± 5.60
C20:4	31.05 ± 14.04	13.54 ± 2.76
C20:0	19.71 ± 21.51	7.17 ± 2.17
C22:0	1.99 ± 1.73	18.92 ± 12.42
C24:0	7.00 ± 7.57	4.30 ± 3.73
Sum of FFAs	2212.54 ± 1445.57	1706.35 ± 417.06

There were no statistically significant differences in fatty acid concentrations (ANOVA, Tukey HSD test, *p* > 0.05). FFA—free fatty acid, SD—standard deviation, ND—not detected.

## Data Availability

The raw data supporting the conclusions of this article will be made available by the authors on request.
